# A Bibliometric Analysis of COVID-19 Research Activity: A Call for Increased Output

**DOI:** 10.7759/cureus.7357

**Published:** 2020-03-21

**Authors:** Mohamad Chahrour, Sahar Assi, Michael Bejjani, Ali A Nasrallah, Hamza Salhab, Mohamad Fares, Hussein H Khachfe

**Affiliations:** 1 Surgery, American University of Beirut Medical Center, Beirut, LBN; 2 Miscellaneous, American University of Beirut Medical Center, Beirut, LBN; 3 Urology, American University of Beirut Medical Center, Beirut , LBN; 4 Sports Medicine, American University of Beirut Medical Center, Beirut, LBN; 5 General Surgery, American University of Beirut Medical Center, Beirut, LBN

**Keywords:** covid19, novel coronavirus, coronavirus disease, bibliometric, public health

## Abstract

Background: The novel coronavirus disease 2019 (COVID-19) has impacted many countries across all inhabited continents, and is now considered a global pandemic, due to its high rate of infectivity. Research related to this disease is pivotal for assessing pathogenic characteristics and formulating therapeutic strategies. The aim of this paper is to explore the activity and trends of COVID-19 research since its outbreak in December 2019.

Methods: We explored the PubMed database and the World Health Organization (WHO) database for publications pertaining to COVID-19 since December 2019 up until March 18, 2020. Only relevant observational and interventional studies were included in our study. Data on COVID-19 incidence were extracted from the WHO situation reports. Research output was assessed with respect to gross domestic product (GDP) and population of each country.

Results: Only 564 publications met our inclusion criteria. These articles came from 39 different countries, constituting 24% of all affected countries. China produced the greatest number of publications with 377 publications (67%). With respect to continental research activity, Asian countries had the highest research activity with 434 original publications (77%). In terms of publications per million persons (PPMPs), Singapore had the highest number of publications with 1.069 PPMPs. In terms of publications per billion-dollar GDP, Mauritius ranked first with 0.075.

Conclusion: COVID-19 is a major disease that has impacted international public health on a global level. Observational studies and therapeutic trials pertaining to COVID-19 are essential for assessing pathogenic characteristics and developing novel treatment options.

## Introduction

The capital of the Chinese province Hebei, Wuhan city, has witnessed starting the 31st of December 2019, the emergence of a new lower respiratory tract disease [1]. Chinese scientists were able to find the virus resulting in the epidemic, which was identified as the severe acute respiratory syndrome coronavirus 2 (SARS-CoV-2), later identified as the COVID-19 [2]. In March 2020, the World Health Organization (WHO) has declared COVID-19 to be a pandemic, with the virus infecting more than 150,000 persons in 154 countries as of the 15th of March [3-4].

Between January and March 2020, worldwide efforts have been focused on dealing with this emerging pandemic [5]. The high infectivity rate of the virus has been a problem in countries where healthcare facilities became saturated and not able to accommodate patients [6]. Case-fatality rate among COVID-19 confirmed cases is estimated to be between 0.8% and 4.2% with the majority of deaths typically occurring among the elderly (age > 80 years), immunocompromised, or those with multiple comorbidities such as cardiovascular disease [7-8]. Until now, the WHO’s and the US Centers for Disease Control and Prevention’s (CDC) directives for the management of COVID-19 have been limited to infection control and symptomatic management of patients. No antiviral drugs or vaccines are available for the coronavirus [9]. Physicians are using different antivirals and anti-inflammatory agents based on expert opinion, as well as case series, and prospective and randomized trials reported from all over the world [10].

That mentioned, research done on COVID-19 is of major importance for both the containment of the disease and the treatment of the patients [11]. Reports from countries with big numbers of confirmed cases would delineate risk factors, clinical features as well as treatment strategies for patients with COVID-19 [12]. This paper aims at exploring the activity and trend of COVID-19 research worldwide since its outbreak in December 2019.

## Materials and methods

The PubMed database of the National Center for Biotechnology Information (NCBI) and WHO database were used to find the publications related to this study. In PubMed, publications were identified by searching for the terms “novel coronavirus 2019,” “coronavirus 2019,” “COVID 2019,” and “COVID 19” in the search field. All publications between December 2019 and March 16, 2020 were included.

Data were also extracted from the WHO database of publications on COVID-19, which is collected from bibliographic databases, table of contents of relevant journals, and other relevant scientific articles. PubMed publications were cross-referenced to the ones found in the WHO spreadsheet on COVID-19 publications. All the relevant articles not present on PubMed were then added to our database. News reports that were present in the WHO database were identified by two reviewers, and were removed from our final database.

Four reviewers (from the authors) looked at the publications. The corresponding author’s country of origin was identified. The publication type was identified and only original articles and case reports were included in our study. The types of studies included basic science studies, epidemiological studies, randomized control trials, prospective trials, retrospective studies, and case series and reports. Descriptive analysis was done to report the number and type of articles from each country. The number of articles in each country was then compared to its number of confirmed cases to identify countries where more publications are needed.

Data from the WHO Health Emergency Dashboard novel COVID-19 situation reports were extracted, from its initiation (January 21, 2020) up until the most recent one (March 18, 2020) [13]. This information was used to compare research output per total number of cases in the country. To avoid bias, we divided the total number of publications by million persons in the country and by billion US dollar of GDP.

## Results

**Figure 1:**
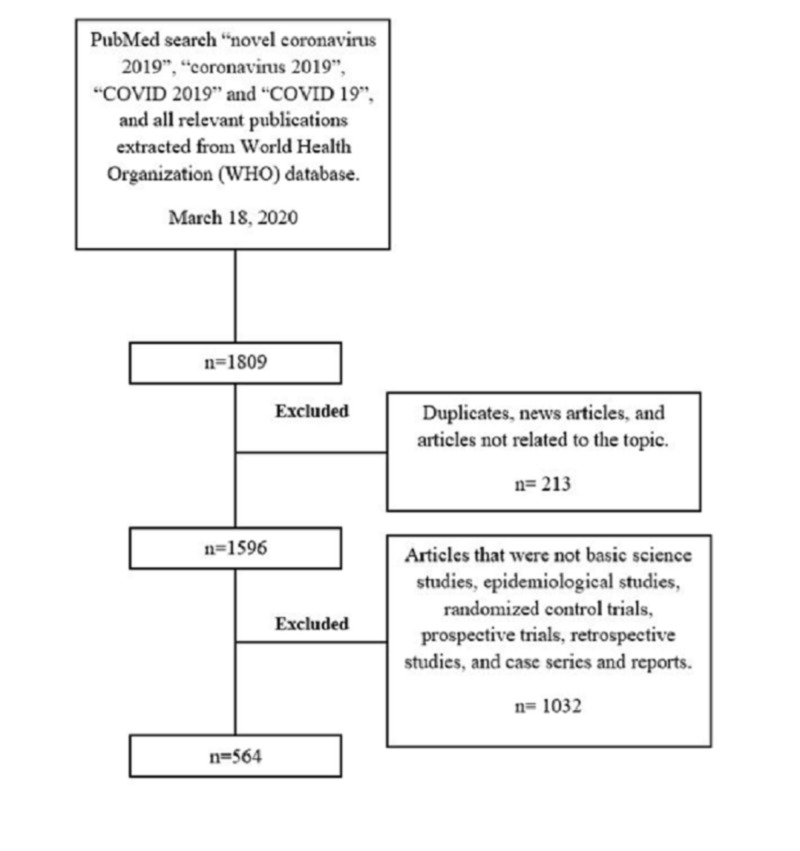
Search strategy and extraction of data.

Our search yielded 1809 publications between December 16, 2019 and March 16, 2020. After removing duplicates, news articles, and articles not related to the topic as deemed by two independent reviewers, we selected 1596 publications for our analysis. Of these, only 564 publications (35%) met our inclusion criteria (Figure 1). The main type of study was “case series” with 247 (43%) publications. There were only five randomized control trials (RCTs) among the original articles group accounting for around 0.8%.

**Figure 2:**
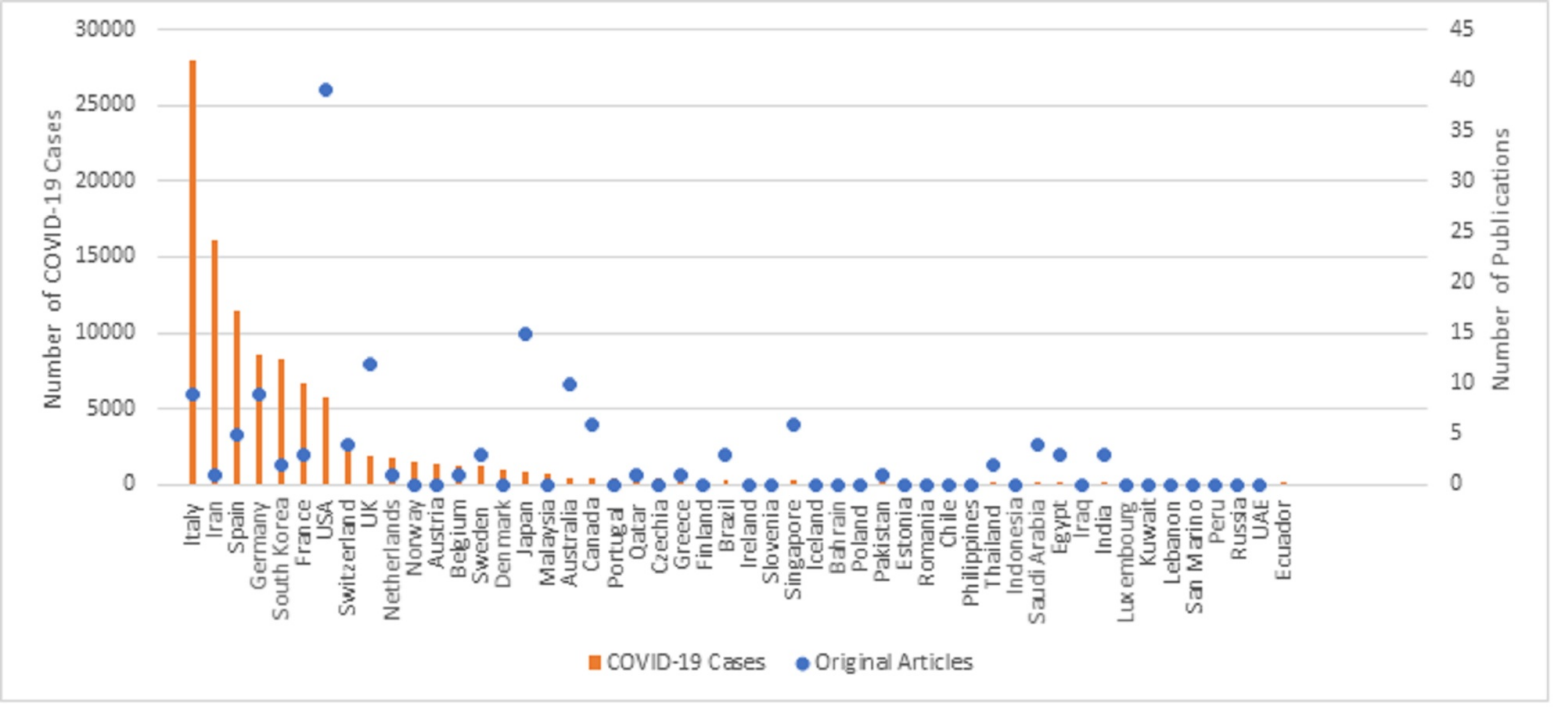
Distribution of novel COVID-19 research output among countries (excluding China) with more than 100 documented cases as of March 18, 2020. COVID-19, coronavirus disease 2019

The 564 studies came from 39 different countries (Table 1). These 39 countries constitute 24% of countries affected by COVID-19. China was the country that produced the greatest number of studies with 377 publications (67%), followed by the United States with 39 publications (7%). Around 17 countries had only one relevant publication on COVID-19 from our search. In terms of continents, Asian countries produced the greatest number with 434 publications (77%), while African and South American countries both ranked last with only six publications (around 1%) each (Table 1). From the countries with over 100 confirmed cases of COVID-19, only 25 countries had published research on COVID-19 (Figure 2).

**Table 1:** Distribution of novel COVID-19 research across countries, and the ratio of the research output to GDP and populations estimate since January 2020. COVID-19, coronavirus disease 2019; GDP, gross domestic product; PPMPs, publication per 1 million persons

Region	Country	Number of publications	PPMPS	Per GDP	Region	Country	Number of publications	PPMPs	Per GDP
Asia	Bangladesh	1	0.006	0.004	Europe	Belgium	1	0.002	0.002
	China	377	0.272	0.031		France	3	0.045	0.001
	India	3	0.002	0.007		Germany	9	0.109	0.002
	Iran	1	0.012	0.002		Greece	1	0.093	0.005
	Japan	15	0.118	0.003		Hungary	1	0.102	0.007
	Korea (North)	17	0.667	-		Italy	9	0.149	0.005
	Nepal	1	0.034	0.041		Netherlands	1	0.034	0.001
	Pakistan	1	0.005	0.003		Spain	5	0.107	0.004
	Qatar	1	0.379	0.006		Sweden	3	0.298	0.006
	Saudi Arabia	4	0.121	0.006		Switzerland	4	0.472	0.006
	Singapore	6	1.069	0.019		United Kingdom	12	0.182	0.005
	South Korea	2	0.039	0.001		Ukraine	1	0.022	0.009
	Taiwan	9	-	-	North America	Canada	6	0.163	0.004
	Thailand	2	0.029	0.004		Mexico	1	0.008	0.001
	Vietnam	2	0.021	0.009		United States of America	39	0.12	0.002
Africa	Egypt	3	0.031	0.013	Oceania	Australia	10	0.407	0.008
	Mauritius	1	0.791	0.075		New Zealand	1	0.209	0.005
	Morocco	1	0.028	0.009	South America	Brazil	3	0.014	0.001
	South Africa	1	0.018	0.003		Columbia	1	0.02	0.003
						Honduras	1	0.108	0.044

In terms of publications per million person (PPMPs), Singapore ranked first with 1.069 PPMPs, followed by Mauritius with 0.791 PPMPs (Table 1). In terms of publications per billion-dollar GDP, Mauritius ranked first with 0.075, followed by Honduras with 0.044 (Table 1).

## Discussion

Number of studies

COVID-19 has arguably been the main focus of medical and scientific institutions around the globe for the past few months, and that is evident by the release of 1596 publications pertaining to it since December 16, 2019. Nevertheless, only around 35% of those constitute observational and interventional studies. China alone has contributed over 66% of the 564 publications, followed by the United States with 39 publications, or almost 7% of the total. For a topic of the utmost medical importance, we may expect to have a larger number of original publications; including but not limited to randomized trials and case series. With the rapid spread of COVID-19 around the globe, healthcare systems and experts were required to mount a rapid response as well. As clinical interventions and contingency planning rose in priority [14], the time and effort needed to write and successfully publish articles became of secondary importance during the acute stages of the pandemic [15].

Over the course of history, humanity has faced a plethora of deadly diseases. From smallpox and the bubonic plague, to AIDS and Ebola, the human race is no stranger to global pandemics [16-18]. As medicine and science advanced, pandemics became less frequent and mortality rates better controlled. The scientific community may be geared towards developing potential vaccines and treatments rather than dedicating valuable time towards observational studies [19]. Conducting RCTs is a lengthy process and publishing results may require several weeks to months; hence, the low volume of therapy-directed research when compared to observational studies is reasonable at this stage [20].

Countries with abundance of cases

The countries with the largest number of cases are China, Italy, Iran, Spain, and Germany in descending order. Considering the fact that China houses over 3.61 million licensed physicians, and is the birthplace of the current pandemic [21], it is not surprising that the majority of relevant publications are attributed to Chinese institutions. However, it is notable that Italy and Iran have produced a combined total of only 10 studies, even though they account for almost 25% of all reported cases. A reason for this may be the strain on the healthcare systems in these said countries; as medical professionals race to keep up with the increasing number of reported cases. Looking at the volume of annual research output per country of 2018, it was found that Italy and Iran ranked 6th and 16th respectively in medical research output, with a combined number of studies close to 60,000 [22]. This further indicates that the research shortage is not due to a lack of research culture or proper academic institutions, but rather due to the overstrain of healthcare facilities and physicians in the face of the rapid disease outbreak. Consequently, patient healthcare constituted a priority over publishing observational studies and case reports.

Of the current 54 countries with 100 or more reported COVID-19 cases, we notice that only five countries have published 10 or more articles. With limited knowledge of this novel virus, the value of case-series and observational studies in countries with a large number of cases should not be underestimated; hence, medical institutions should be contacted and encouraged to publish their findings. Population-level data can be a valuable tool for designing medical management algorithms and guidelines.

Value of observational and interventional studies

As history can attest to, humanity has been surviving epidemics with improved outcomes. This is largely due to our most important line of defense, information. The quicker quality information can be gathered about a newly arising disease, the earlier scientific experts and researchers would be able to procure novel treatments, and ideally, cures. This information begins with early-outbreak case reports and observational studies, where the basic characteristics of the novel disease are documented, and hence, awareness can be raised [23]. Furthermore, randomized controlled trials of currently available antivirals and/or immune modifiers may procure valuable data, useful for treating severe cases and limiting morbidity. In what appears to be an arms-race between pathogens and the medical community, the importance of observational and interventional research cannot be understated [24-26]. In the COVID-19 era, evidence-based medicine can generate evidence-based survival. Thus, we recommend increasing research output, from all countries involved with the disease, to better understand its pathogenic characteristics and help find proper therapeutic modalities.

To the best of our knowledge, this is the first bibliometric analysis to study the worldwide COVID-19 research output. Nevertheless, there are some limitations in our study. Even though we made use of both PubMed and the WHO databases, there still might be publications that were not in our scope. We were also dependent on the indexing of the databases used, as is the case in any other bibliometric study.

## Conclusions

The outbreak of COVID-19 has caused a major threat to the international community and has raised significant public health concerns. The wide spread of this disease, along with its high rate of infectivity has incited a global demand for relevant research that can help describe the clinical and pathogenic characteristics of this illness. Observational studies related to COVID-19 can help describe the symptomatology of the disease, assess the efficiency of diagnostic tools, and establish proper management guidelines. Therapeutic trials can help discover novel treatments and come up with new curative options. Raising these concerns is essential for increasing the global research output pertaining to COVID-19.
